# Altered Bioavailability of Nitric Oxide and L-Arginine Is a Key Determinant of Endothelial Dysfunction in Preeclampsia

**DOI:** 10.1155/2020/3251956

**Published:** 2020-10-22

**Authors:** Worlanyo Tashie, Linda Ahenkorah Fondjo, William K. B. A. Owiredu, Richard K. D. Ephraim, Listowell Asare, Enoch Appiah Adu-Gyamfi, Laila Seidu

**Affiliations:** ^1^Department of Molecular Medicine, SMS, KNUST, Ghana; ^2^Department of Medical Laboratory Technology, University of Cape Coast, Ghana; ^3^Comboni Hospital, Ho, Volta Region, Ghana; ^4^Department of Physiology, University of Cape Coast, Ghana

## Abstract

**Background:**

Preeclampsia is a major cause of maternal and neonatal morbidity and mortality in sub-Saharan Africa. Evidence indicates that endothelial dysfunction is central to the pathogenesis of preeclampsia. This study assessed the level of the components of the arginine-nitric oxide pathway to evaluate endothelial dysfunction in normotensive pregnancies and pregnancies complicated with preeclampsia.

**Methods:**

This case-control study was conducted among pregnant women who visited Comboni Hospital from January 2017 to May 2018. A total of 180 pregnant women comprising 88 preeclamptic women (PE) and 92 healthy normotensive pregnant women (NP) were recruited. Sociodemographic, clinical, and obstetric data were obtained using validated questionnaires. Blood pressure and anthropometrics were measured, and blood samples were collected for the estimation of nitric oxide (NO^∙^), L-arginine, asymmetric dimethylarginine (ADMA), and 3-nitrotyrosine using an enzyme-linked immunosorbent assay technique.

**Results:**

The mean NO^∙^ (*p* = 0.010) and L-arginine/ADMA ratio (*p* < 0.0001) was significantly lower in PE compared to NP while mean L-arginine (*p* = 0.034), ADMA (*p* < 0.0001), and 3-nitrotyrosine (*p* < 0.0001) were significantly higher in PE than NP. ADMA showed a significant positive association with systolic blood pressure (*β* = 0.454, *p* = 0.036) in severe PE. Women with PE had significant intrauterine growth restriction (*p* < 0.0001) and low birth weight infants (*p* < 0.0001) when compared to NP.

**Conclusion:**

Preeclampsia is associated with reduced NO^∙^ bioavailability, L-arginine/ADMA ratio, and elevated levels of ADMA and 3-nitrotyrosine. Measurements of the levels of these parameters can help in the early prediction of endothelial dysfunction in preeclampsia. Exogenous therapeutic supplementation with L-arginine during pregnancy to increase the L-arginine/ADMA ratio should be considered to improve endothelial function in preeclampsia and pregnant women at risk of developing preeclampsia.

## 1. Background

Preeclampsia complicates about 2-8% of all pregnancies worldwide [1, 2] and poses a major challenge to both the mother and fetus [3–5]. It accounts for about 10-15% of all maternal deaths in developing countries [1, 2]. In two different regions in Ghana, the incidence of preeclampsia has been reported to be between 6.55% and 7.03% [6, 7]. Despite the several advances made in modern medicine globally, preeclampsia still remains a major challenge in understanding both its precise etiology and management. Evidence, however, suggests that generalized endothelial dysfunction (ED) is central to the pathogenesis of preeclampsia [8, 9]. A dysfunctional endothelium is the common final pathway leading to the clinical signs of this disorder, including hypertension and significant proteinuria [10]. A major contributing factor to the development of vascular endothelial dysfunction is altered bioavailability of nitric oxide (NO^∙^) [11].

**Figure 1:**
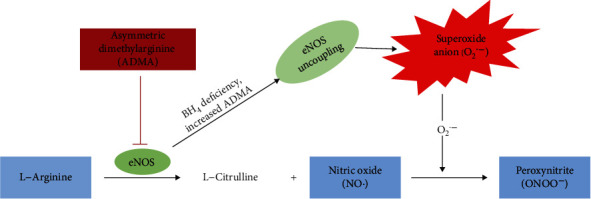
The L-arginine-nitric oxide pathway.

NO^∙^ is a potent signaling molecule that maintains endothelial integrity by regulating vasodilation, adhesion of leucocytes to blood vessels, and platelet aggregation [12]. NO^∙^ is synthesized in every cell type through the action of the enzyme nitric oxide synthase (NOS). [12–14]. All NOS isoforms utilize L-arginine as the precursor for synthesizing NO^∙^ [15–17]. Asymmetric dimethylarginine (ADMA), a methylated product of L-arginine, inhibits all isoforms of NOS endogenously, hence leading to decreased NO^∙^ formation from L-arginine [13]. The L-arginine/ADMA ratio is the ratio of the substrate, L-arginine, to its competitive inhibitor, ADMA [16]. The L-arginine/ADMA ratio may act as a key determinant for NOS function [17]. Furthermore, NO^∙^ can react *in vivo* with superoxide anion (O_2_^·−^) to produce peroxynitrite (ONOO^−^) (Figure 1) [18]. Tyrosine residues react with ONOO^−^ to produce 3-nitrotyrosine, which is extremely useful for measuring the formation of ONOO^−^ within the body [18]. Reduced bioavailability of NO^∙^ in the body could therefore result either from decreased synthesis and/or increased degradation by other molecules, such as reactive oxygen species (ROS). However, reports from studies on levels of NO^∙^ and ADMA in preeclampsia are conflicting as studies have indicated that levels of NO^∙^ and ADMA in maternal plasma, serum, or urine are either the same, higher, or lower in preeclampsia compared to normotensive pregnancy [10, 12]. Moreover, there is sparse data on the potential of these biomarkers of ED in women with preeclampsia from African origin. Likewise, there are no specific biomarkers in routine practice for early screening of high-risk women for ED in preeclampsia, especially in our environment. It is against this background that we hypothesized that altered bioavailability of NO^∙^ and the factors that affect NO^∙^ bioavailability in pregnancy compromises the integrity of the endothelium. This study, therefore, assessed major components of the arginine-NO^∙^ pathway (NO^∙^, L-arginine, ADMA, 3-nitrotyrosine, and L-arginine/ADMA ratio) as possible markers of endothelial dysfunction in normotensive pregnancies and pregnancies complicated with preeclampsia.

## 2. Materials and Methods

### 2.1. Study Design and Study Setting

This case-control study was carried out from January 2017 to May 2018 at the Comboni Hospital, located in the Volta Region of Ghana. The hospital is located at Sogakope in the South Tongu district of the Volta Region with a population of 87,950 (Ghana Statistical Services, 2010). The Comboni Hospital has a 50-bed capacity and provides health services to clients from the Southern part of the Volta Region of Ghana, most especially with referrals from the South Tongu district, North Tongu district, Keta Municipality, and Ketu North district.

### 2.2. Ethical Considerations

The Committee on Human Research, Publications and Ethics (CHRPE), School of Medical Sciences, Kwame Nkrumah University of Science & Technology (KNUST), and the Institutional Review Board of Comboni Hospital approved the study protocol (CHRPE/AP/523/17). Each participant gave a written consent prior to enrolment into this study.

### 2.3. Study Participants

One hundred and eighty (180) pregnant women comprising eighty-eight (88) preeclamptic women and ninety-two (92) healthy normotensive pregnant women and with gestational age > 20 weeks were recruited into this study. The study participants were aged 18 to 40 years. The preeclamptic women were recruited by a qualified obstetrician/gynaecologist using the National High Blood Pressure Education Program Working Group diagnostic criteria [19]. Preeclampsia was diagnosed based on the new onset of hypertension and proteinuria (≥1+ reading on dipstick) occurring after the 20^th^ week of gestation in a previously normotensive and nonproteinuric woman. Pregnant women with blood pressure ≥ 160/110 mmHg and proteinuria (≥3+ reading on dipstick) were classified as having severe preeclampsia while pregnant women with systolic blood pressure (SBP) of 140–159 mmHg, diastolic blood pressure (DBP) of 90–109 mmHg, and proteinuria (≥1+ reading on dipstick) were classified as having mild preeclampsia. The control group comprised of age- and gestation-matched healthy normotensive pregnant women. A detailed sociodemographic, medical, and obstetric data was obtained from each participant using a well-structured validated questionnaire (see Suppl. File 1).

### 2.4. Eligibility Criteria

All recruited participants were singleton pregnant women, aged 18-40 years and with gestational age > 20 weeks. Healthy normotensive pregnant women and pregnant women with a confirmed diagnosis of preeclampsia were included. This study excluded women who failed to give written consent. Pregnant women with twin gestation or history of chronic hypertension, diabetes mellitus, kidney disease, gestational diabetes, or cardiovascular disorders were also excluded.

### 2.5. Blood Pressure Measurement

The blood pressure of each participant was measured in the morning prior to venous blood sample collection. Each participant was asked to sit down comfortably, extend the left arm on a table, and then relax for 10 minutes. An automated Accoson mercury sphygmomanometer (Essex, United Kingdom) was used by a trained personnel to measure the systolic blood pressure and diastolic blood pressure of each participant according to standard guidelines [20]. Blood pressure was measured twice for each participant, 6-15 minutes apart. The mean blood pressure of the two measurements was reported as the blood pressure of each participant.

### 2.6. Determination of Infant Birth Weight and Intrauterine Growth Restriction

Infant birth weight and estimated fetal weight (EFW) were obtained for each participant. All infants were weighed at birth using a weighing scale (Docbel Braun Baby Classic Weighing Scale, New Delhi, India). Neonates who weighed <2.5 kg at birth were considered having low birth weight whereas neonates who weighed ≥2.5 kg at birth were classified as having normal weight. Ultrasound, carried-out in the third trimester of pregnancy, was used to assess EFW from each participant. Using the smoothed percentiles of the EFW for gestational age obtained from ultrasound measurements, IUGR was classified using the 10^th^ percentile (see Suppl. File 2 for smoothed percentiles of estimated fetal weight (grams) for gestational age).

### 2.7. Blood Sample Collection and Biochemical Assay

About eight milliliters (8 mL) of venous blood was drawn from each participant into serum separator test tubes (BD Vacutainer, USA) after 8-12-hour overnight fast. After clotting, the samples were centrifuged at 4000 rpm for 8 minutes. The serum was separated and stored at -20°C until assayed. The stored serum was used to estimate NO^∙^, ADMA, L-arginine, and 3-nitrotyrosine.

NO^∙^, L-arginine, ADMA, and 3-nitrotyrosine were estimated using enzyme-linked immunosorbent assay (ELISA) kits (MyBioSource, Inc., San Diego, California, USA). The optical density was read at 450 nm using Mindray MR-96A microplate reader (Shenzhen, China). The serum level of each potential biomarker in the test samples was calculated from the standard curve plotted using the different standards with known antigen concentration.

### 2.8. Statistical Analysis

Data was analyzed using IBM SPSS (version 25.0. Armonk, NY: IBM Corp) and Microsoft Excel (version 2013). The dataset had no missing data. Continuous data were reported as mean ± standard deviation (SD), while categorical data were reported as proportions and percentages. Comparison of the continuous data was carried out using the independent-sample *t*-test. One-way analysis of variance (ANOVA) and one-way analysis of covariance (ANCOVA) followed by Bonferroni post hoc analysis were used to compare the means between more than two groups. Chi-squared (*χ*^2^) and Fisher's exact tests were used to compare categorical data. The association between components of the arginine-NO^∙^ pathway and the clinical parameters was done using linear regression analysis after adjusting for the confounding factors (maternal age, gestational age, and BMI). Two-tailed tests were used for all analysis. Statistical significance level was considered at *p* < 0.05.

## 3. Results

The clinical variables of the study participants are summarized in Table 1. Both PE and NP presented with similar maternal age (*p* = 0.462) and gestational age at sampling (*p* = 0.104). NO^∙^ levels (*p* = 0.010) and L-arginine/ADMA ratio (*p* < 0.0001) were significantly decreased in PE compared to NP. L-Arginine (*p* = 0.034), ADMA (*p* < 0.0001), and 3-nirotyrosine (*p* < 0.0001) were significantly higher in PE than NP (*p* < 0.05).

**Figure 2:**
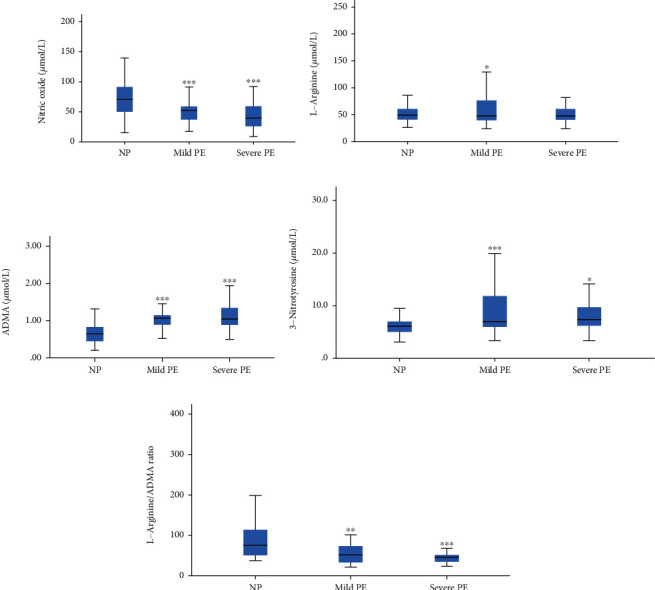
Levels of components of the arginine-NO^∙^ pathway in mild and severe preeclampsia. (a) NO^∙^; (b) L-arginine; (c) ADMA; (d) 3-nitrotyrosine; (e) L-arginine/ADMA ratio. NP: normotensive pregnant women; PE: preeclamptic women; ADMA: asymmetric dimethylarginine. ^∗^*p* < 0.05; ^∗∗^*p* < 0.01; ^∗∗∗^*p* < 0.0001. One-way ANOVA followed by Bonferroni post hoc analysis to compare both mild PE and severe PE to NP.

Figure 2 depicts the components of the arginine-NO^∙^ pathway of the study participants stratified by the severity of preeclampsia. About sixty-four percent (63.6%) of women presenting with PE had mild preeclampsia whereas 36.4% had severe preeclampsia. The levels of NO^∙^ and L-arginine/ADMA ratio were significantly decreased in the order of NP > mild PE > severe PE (*p* < 0.05), while the level of ADMA was significantly elevated in the order of NP < mild PE < severe PE (*p* < 0.05). The difference in L-arginine levels between NP and severe PE was insignificant (*p* > 0.05) while showing a significant difference between NP and mild PE (*p* < 0.05). Although the levels of 3-nitrotyrosine in mild and severe PE were significantly higher than those in NP (*p* < 0.05), the levels were elevated in mild PE than in severe PE.

Table 2 summarizes the infant birth weight and IUGR of the study participants. The infant birth weight in PE was significantly lower in comparison to NP (*p* < 0.0001). About fifty-three percent (53.4%) of the women presenting with PE had low birth weight babies whereas 9.8% of NP had low birth weight babies. While none of the NP had IUGR, growth restriction was demonstrated in 37.5% of PE cases. About forty-nine percent (48.9%) of the women with PE had preterm delivery compared to 3.3% in NP, and this was statistically significant (*p* < 0.0001).

**Figure 3:**
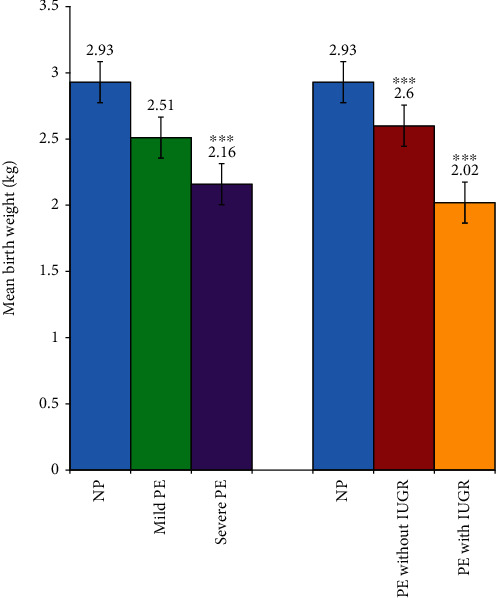
Birth weight of babies stratified by disease severity and complication by IUGR after adjusting for gestational age at delivery. NP: normotensive pregnant women; PE: preeclamptic women; IUGR: intrauterine growth restriction. ^∗∗∗^*p* < 0.0001. One-way ANCOVA followed by Bonferroni post hoc analysis to compare the groups with NP after adjusting for gestational age at delivery.

Figure 3 depicts the infant birth weight in the study participants stratified by disease severity and complication by IUGR after adjusting for gestational age at delivery. Babies from mothers with mild and severe preeclampsia were associated with significantly lower birth weight compared to those from NP (*p* < 0.0001). Likewise, between the preeclampsia groups, babies from mothers with severe PE had lower birth weight compared to those from mild PE. Also, both PE with IUGR and PE without IUGR had significantly lower birth weight neonates in comparison to NP (*p* < 0.0001).

Table 3 summarizes the linear regression analysis of components of the arginine-NO^∙^ pathway with blood pressure. Since hypertension is a key clinical manifestation of preeclampsia, systolic blood pressure and diastolic blood pressure were taken as a dependent variable, with NO^∙^, L-arginine, ADMA, 3-nitrotyrosine, and L-arginine/ADMA ratio as independent variables of risk estimate. ADMA showed a positive association with SBP (*β* = 0.454, *p* = 0.009) in severe preeclampsia.

## 4. Discussion

This study demonstrated a significant reduction in the level of NO^∙^ with a corresponding significant increase in levels of L-arginine, ADMA, and 3-nitrotyrosine in pregnant women presenting with preeclampsia compared to normotensive pregnancy (Table 1). While both L-arginine and ADMA were significantly elevated in preeclampsia compared to normotensive pregnancy, the L-arginine/ADMA ratio in the preeclamptic women was significantly reduced. Reduced bioavailability of endothelial-derived NO^∙^ is one of the first indications of endothelial dysfunction and has been linked to severe vascular pathologies [21]. There are contradictory reports on the levels of NO^∙^ in relation to preeclampsia. The decreased NO^∙^ levels observed in women with preeclampsia in our study agrees with the findings of some similar studies [22, 23]. Conversely, other studies observed significantly increased NO^∙^ levels in preeclampsia compared to normal pregnancy [9, 24, 25], while others observed no change in NO^∙^ levels compared to normal pregnancy [26, 27]. Particularly, a study conducted in Ghana by Adu-Bonsaffoh et al. [9] observed an elevated NO^∙^ level in preeclampsia compared to normotensive pregnancy. The authors attributed the elevated NO^∙^ levels to a dysregulated compensatory reaction to restore the generalized endothelial damage [9]. Our present study, however, did not only evaluate NO^∙^ levels but also assessed the possible factors that could affect NO^∙^ synthesis. It is likely that the divergent findings of the other studies may be attributed to the variances in the methods used in NO^∙^ estimation, disease severity in the various study groups, and the gestational age at which NO^∙^ was evaluated. However, it has been demonstrated that the concentrations of cyclic GMP are consistently reduced in preeclampsia, suggesting decreased NO^∙^ bioactivity in preeclampsia [28].

The low NO^∙^ levels observed in preeclampsia compared to healthy normotensive pregnancy in this study could be as a result of the endogenous inhibition of eNOS by ADMA. Consistent with previous findings [17, 23, 29–32], this study reports elevated ADMA in preeclamptic women (Table 1). Other studies [33–35], however, observed unchanged ADMA levels in preeclamptic women compared to normotensive pregnancy. It is noteworthy that at optimal concentration, ADMA has very minimal effects on eNOS activity. However, an elevated level of ADMA is capable of inhibiting the activity of eNOS [16]. Increased shear stress triggers ADMA synthesis, and elevated ADMA in hypertension may therefore be an epiphenomenon of high blood pressure [36].

L-Arginine bioavailability is a key determinant of NO^∙^ formation in the body [16]. There is optimal synthesis of NO^∙^ at a physiological level of L-arginine. Interestingly, from this study, the level of L-arginine in both the preeclamptic and normotensive pregnant women were within physiologic limits (Table 1). However, women with preeclampsia had significantly higher L-arginine concentration with correspondingly decreased NO^∙^ levels compared to normotensive pregnant women. Conversely, other studies [33, 37] observed decreased serum L-arginine levels in preeclampsia. The low NO^∙^ concentration in preeclampsia in spite of adequate L-arginine concentration from this study could be due to the defective transport of L-arginine via the y^+^ transport system [36] or as result of the observed elevated ADMA levels, which competitively inhibit the cellular uptake of L-arginine via the y+ transport system [16].

Furthermore, findings of this study showed increased ONOO^−^ activity and oxidative stress in preeclampsia as evidenced by elevated 3-nitrotyrosine concentrations in preeclampsia (Table 1). In agreement with this study, Noris and his colleagues [38] demonstrated an excess formation of ONOO^−^ in a preeclamptic placenta. ONOO^−^ formation does not only inactivate NO^∙^ but also leads to uncoupling of eNOS [39]. Uncoupled eNOS generates O_2_^·−^ rather than NO^∙^ [40].

It is logical to interpret L-arginine concentrations in relation to the concentrations of ADMA, a competitive inhibitor of eNOS activity. Since ADMA competes with L-arginine for eNOS, one potential consideration on the net effect of ADMA in the synthesis of NO^∙^ will be the balance between L-arginine and ADMA. Thus, an imbalance between L-arginine and ADMA will result in relative L-arginine deficiency. The L-arginine/ADMA ratio, therefore, acts as a key determinant for NOS function than ADMA or L-arginine alone and may serve as a better marker in assessing endothelial function than ADMA or L-arginine alone. Similar to the observations of Luneburg et al. [17], this study also demonstrated a significant decrease in L-arginine/ADMA ratio in preeclampsia compared to normal pregnancy (Table 1). In line with the findings of Isik and colleagues [41], the decrease in L-arginine/ADMA ratio in preeclamptic women in this study suggests impairment in NO^∙^ synthesis. At physiological concentration of both L-arginine and ADMA, NOS is well-saturated with L-arginine such that there is optimal production of NO^∙^. Under such conditions, L-arginine supplementation does not alter eNOS activity [16]. Meanwhile, pathological levels of ADMA decreases eNOS activity and subsequently leads to the decreased synthesis of NO^∙^. Subsequent to other studies [16, 17], we agree that, in the presence of elevated ADMA, the exogenous supply of L-arginine could replace the competitive inhibitor and restore the L-arginine/ADMA ratio to a level that promotes eNOS activity. Some studies have indicated the essential role of exogenous L-arginine supplementation in enhancing NO^∙^ synthesis [42, 43]. A number of mechanisms have been proposed to explain the role of exogenous L-arginine supplementation in evoking beneficial effects in improving endothelial function. One possible mechanism is linked to the rate of L-arginine transport and not to its intracellular levels [16]. L-Arginine is compartmentalized within cells, and it has been shown that there is colocalization of eNOS and the L-arginine transporter CAT-1 in specialized membrane regions called caveolae of endothelial cells [16]. It is possible that this colocalization allows for direct delivery of extracellular arginine to eNOS and less accessible to intracellular L-arginine [42–44]. The findings of these studies suggest that L-arginine transported via CAT-1 may not be in rapid equilibrium with intracellular L-arginine and that extracellular L-arginine may be essential in promoting NO^∙^ synthesis by endothelial cells [43].

Our study showed that the alteration in NO^∙^, ADMA, and L-arginine/ADMA ratio in preeclamptic women compared to the normal pregnant women worsens with increasing disease severity (Figure 2). Likewise, Ellis et al. [29] observed that ADMA levels were significantly higher in subjects with mild and severe preeclampsia compared to normal pregnant women. Contrary to the findings of Chamy et al. [45], our study showed that there is marked oxidative stress in mild preeclampsia compared to severe preeclampsia as evidenced by the 3-nitrotyrosine concentration in this study. This could be as a result of compensatory mechanism in the women with severe preeclampsia.

Additionally, our present study demonstrates a significant positive association of ADMA with systolic blood pressure in severe preeclampsia (Table 3), which agrees with the findings of Zheng and colleagues [46]. In another study by Holden et al. [31], it was also reported that the early fall in blood pressure in normotensive pregnancy is accompanied by a significant reduction in the serum ADMA levels, suggesting that elevated levels of ADMA may play major roles in regulating maternal vascular dilatation and blood pressure. Thus, serum ADMA levels may predict preeclampsia, even before the occurrence of hypertension and proteinuria and hence could be used for assessing a possible rise in blood pressure and future occurrence of preeclampsia.

In agreement with these studies [47–51], we demonstrated that women with preeclampsia presented with complications such as IUGR and adverse birth outcomes such as preterm delivery and low birth weight neonates (Table 2 and Figure 3), implying that preeclampsia still remains a major cause of adverse pregnancy outcomes in our environment.

The expression of eNOS was not assessed in our study which is a major limitation of this study. Nevertheless, the findings of this study contribute significantly to scientific knowledge.

## 5. Conclusion

Preeclampsia is associated with reduced NO^∙^ bioavailability and L-arginine/ADMA ratio and elevated levels of ADMA and 3-nitrotyrosine. Measurements of these parameters can help in the early prediction of endothelial dysfunction in preeclampsia. Elevated ADMA levels may precede hypertension and proteinuria and hence could be used for predicting preeclampsia. Preeclampsia predisposes women to having preterm delivery, IUGR, and low birth weight babies. Exogenous supplementation with L-arginine during pregnancy could be explored as a therapeutic agent to increase the L-arginine/ADMA ratio and improve endothelial function in preeclampsia and pregnant women at risk of developing preeclampsia.

## Figures and Tables

**Table 1:** Clinical and biochemical variables of the studied participants.

Parameters	Total (*n* = 180)	NP (*n* = 92)	PE (*n* = 88)	*p* value
Age (years)	29.00 ± 4.84	28.74 ± 4.95	29.27 ± 4.74	0.462
GA at sampling (weeks)	31.24 ± 3.29	30.85 ± 3.08	31.65 ± 3.48	0.104
Nitric oxide (*μ*mol/L)	65.81 ± 32.89	78.34 ± 35.82	52.70 ± 23.33	0.010
L-Arginine (*μ*mol/L)	57.51 ± 26.85	53.29 ± 14.93	61.92 ± 34.80	0.034
ADMA (*μ*mol/L)	0.89 ± 0.40	0.67 ± 0.27	1.12 ± 0.38	<0.0001
3-Nitrotyrosine (mmol/L)	8.00 ± 4.47	6.52 ± 2.51	9.54 ± 5.46	<0.0001
L-Arginine/ADMA ratio	79.44 ± 56.12	97.90 ± 61.151	60.14 ± 42.78	<0.0001

PE: preeclamptic women; NP: normotensive pregnant women; GA: gestational age; BMI: body mass index; ADMA: asymmetric dimethylarginine. Data was presented as mean ± SD. The means of the clinical variables in PE were compared with NP using independent *t*-test. Statistical significance level was considered at *p* value < 0.05.

**Table 2:** Pregnancy characteristics and birth outcomes in normal pregnancy and preeclampsia.

Variables	Total (*n* = 180)	NP (*n* = 92)	PE (*n* = 88)	*p* value
Birth weight (kg)	2.66 ± 0.47	2.93 ± 0.38	2.38 ± 0.40	<0.0001
*Birth weight*				
Normal birth weight	124 (68.9)	83 (90.2)	41 (46.6)	<0.0001
Low birth weight	56 (31.1)	9 (9.8)	47 (53.4)	
*IUGR*				
Yes	33 (18.3)	0 (0.0)	33 (37.5)	<0.0001
No	147 (81.7)	100 (100.0)	55 (62.5)	
*Status of delivery*				<0.0001
Term	134 (74.4)	89 (96.7)	45 (51.1)	
Preterm	46 (25.6)	3 (3.3)	43 (48.9)	

PE: preeclamptic women; NP: normotensive pregnant women; IUGR: intrauterine growth restriction. Continuous data was presented as mean ± SD, while categorical data was expressed as frequency (percentage). Independent *t*-test was used to compare means of the continuous data while *χ*^2^ or Fischer's exact test (when *n* < 5 was used to compare the categorical data. Statistically significant level was set at a *p* value < 0.05.

**Table 3:** Linear regression analysis of potential biomarkers associated with the risk of preeclampsia after adjusting for maternal age, BMI, and gestational age.

Dependent variable	Parameter	Mild preeclampsia		Severe preeclampsia	
		*β* coefficient	*p* value	*β* coefficient	*p* value
Systolic blood pressure	Nitric oxide (*μ*mol/L)	-0.160	0.214	0.313	0.106
	L-Arginine (*μ*mol/L)	-0.034	0.787	0.181	0.341
	ADMA (*μ*mol/L)	0.137	0.316	0.454	0.036
	3-Nitrotyrosine (mmol/L)	-0.165	0.195	0.303	0.128
	L-Arginine/ADMA ratio	-0.052	0.695	-0.066	0.741

Diastolic blood pressure	Nitric oxide (*μ*mol/L)	-0.153	0.263	0.247	0.128
	L-Arginine (*μ*mol/L)	-0.204	0.132	0.123	0.443
	ADMA (*μ*mol/L)	-0.084	0.564	0.304	0.098
	3-Nitrotyrosine (mmol/L)	-0.171	0.207	0.253	0.129
	L-Arginine/ADMA ratio	-0.155	0.269	-0.005	0.977

ADMA: asymmetric dimethylarginine. Statistically significant level was set at a *p* value < 0.05.

## Data Availability

All data set used or analysed or generated during the current study are included in this article and its supplementary files.

## Supplementary Materials

Supplementary 1 Research questionnaire.

Supplementary 2 Smoothed percentiles of estimated birth weight (grams) for gestational age [52].

## References

[1] Duley L. (2009). The global impact of pre-eclampsia and eclampsia. *Seminars in Perinatology*.

[2] Salam R., Das J., Ali A., Bhaumik S., Lassi Z. (2015). Diagnosis and management of preeclampsia in community settings in low and middle-income countries. *Journal of Family Medicine and Primary Care*.

[3] Mustafa R., Ahmed S., Gupta A., Venuto R. C. (2012). A comprehensive review of hypertension in pregnancy. *Journal of Pregnancy*.

[4] Zhou A., Xiong C., Hu R. (2015). Pre-pregnancy BMI, gestational weight gain, and the risk of hypertensive disorders of pregnancy: a cohort study in Wuhan, China. *PLoS One*.

[5] Fondjo L. A., Gyamfi E. A. A., Owiredu W. K. B. A., Turpin C. A., Mante D. A., Anto E. O. (2016). Maternal serum adiponectin, leptin and adiponectin-leptin ratio as possible biomarkers of preeclampsia. *Edorium Journal of Gynecology and Obstetrics*.

[6] Obed S., Aniteye P. (2006). Birth weight and ponderal index in pre-eclampsia: a comparative study. *Ghana Medical Journal*.

[7] Ahenkorah L. (2009). *Metabolic syndrome, oxidative stress and putative risk factors amongst Ghanaian women presenting with pregnancy-induced hypertension*.

[8] Chambers J. C., Fusi L., Malik I. S., Haskard D. O., De Swiet M., Kooner J. S. (2001). Association of maternal endothelial dysfunction with preeclampsia. *Journal of the American Medical Association*.

[9] Adu-Bonsaffoh K., Antwi D. A., Obed S. A., Gyan B. (2015). Nitric oxide dysregulation in the pathogenesis of preeclampsia among Ghanaian women. *Integrated Blood Pressure Control*.

[10] Braekke K., Ueland P. M., Harsem N. K., Staff A. C. (2009). Asymmetric Dimethylarginine in the maternal and fetal circulation in preeclampsia. *Pediatric Research*.

[11] Rytlewski K., Olszanecki R., Korbut R., Zdebski Z. (2005). Effects of prolonged oral supplementation with L-arginine on blood pressure and nitric oxide synthesis in preeclampsia. *European Journal of Clinical Investigation*.

[12] Sánchez-Aranguren L. C., Prada C. E., Riaño-Medina C. E., Lopez M. (2014). Endothelial dysfunction and preeclampsia: role of oxidative stress. *Frontiers in Physiology*.

[13] Huang L.-T., Hsieh C.-S., Chang K.-A., Tain Y.-L. (2012). Roles of nitric oxide and asymmetric dimethylarginine in pregnancy and fetal programming. *International Journal of Molecular Sciences*.

[14] Cai H., Harrison D. G. (2000). Endothelial dysfunction in cardiovascular diseases the role of oxidant stress. *Circulation Research*.

[15] Forstermann U., Sessa W. C. (2012). Nitric oxide synthases: regulation and function. *European Heart Journal*.

[16] Celik M., Unal H. M., Patel V. B., Preedy V. R., Rajendram R. (2017). The l-arginine/asymmetric dimethylarginine (ADMA) ratio in health and disease: an overview. *L-Arginine in Clinical Nutrition*.

[17] Luneburg N., Xanthakis V., Schwedhelm E. (2011). Reference intervals for plasma L-arginine and the L-arginine:asymmetric dimethylarginine ratio in the Framingham Offspring Cohort. *The Journal of Nutrition*.

[18] Lopez-Jaramillo P., Arenas W. D., Garcia R. G., Rincon M. Y., Lopez M. (2008). Review: the role of the L-arginine-nitric oxide pathway in preeclampsia. *Therapeutic Advances in Cardiovascular Disease*.

[19] ACOG practice bulletin (2002). Diagnosis and management of preeclampsia and eclampsia. *International Journal of Gynaecology and Obstetrics*.

[20] Pickering T. G., Hall J. E., Appel L. J. (2005). Recommendations for blood pressure measurement in humans and experimental animals. *Hypertension*.

[21] Hadi H. A. R., Carr C. S., Al-Suwaidi J. (2005). Endothelial dysfunction: cardiovascular risk factors, therapy, and outcome. *Vascular Health and Risk Management*.

[22] Choi J. W., Im M. W., Pai S. H. (2002). Nitric oxide production increases during normal pregnancy and decreases in preeclampsia. *Annals of Clinical and Laboratory Science*.

[23] Mao D., Che J., Li K. (2010). Association of homocysteine, asymmetric dimethylarginine, and nitric oxide with preeclampsia. *Archives of Gynecology and Obstetrics*.

[24] Bartha J. L., Comino-Delgado R., Bedoya F. J., Barahona M., Lubian D., Garcia-Benasach F. (1999). Maternal serum nitric oxide levels associated with biochemical and clinical parameters in hypertension in pregnancy. *European Journal of Obstetrics & Gynecology and Reproductive Biology*.

[25] Shetty S., Rai T. S., Ullal H., Shetty S. (2016). A study on the levels of nitric oxide and lipid peroxides in pre-eclampsia. *International Journal of Recent Scientific Research*.

[26] Darkwa E. O., Djagbletey R., Essuman R., Sottie D., Dankwah G. B., Aryee G. (2018). Nitric oxide and pre-eclampsia: a comparative study in Ghana. *Open Access Macedonian Journal of Medical Sciences*.

[27] Hata T., Hashimoto M., Kanenishi K., Akiyama M., Yanagihara T., Masumura S. (1999). Maternal circulating nitrite levels are decreased in both normal normotensive pregnancies and pregnancies with preeclampsia. *Gynecologic and Obstetric Investigation*.

[28] Moonen R. M., Huizing M. J., Cavallaro G. (2015). Plasma levels of dimethylarginines in preterm very low birth weight neonates: its relation with perinatal factors and short-term outcome. *International Journal of Molecular Sciences*.

[29] Ellis J., Wennerholm U. B., Bengtsson A. (2001). Levels of dimethylarginines and cytokines in mild and severe preeclampsia. *Acta obstetricia et gynecologica Scandinavica*.

[30] Pettersson A., Hedner T., Milsom I. (1998). Increased circulating concentrations of asymmetric dimethyl arginine (ADMA), an endogenous inhibitor of nitric oxide synthesis, in preeclampsia. *Acta obstetricia et gynecologica Scandinavica*.

[31] Holden D. P., Fickling S. A., Whitley G. S., Nussey S. S. (1998). Plasma concentrations of asymmetric dimethylarginine, a natural inhibitor of nitric oxide synthase, in normal pregnancy and preeclampsia. *American Journal of Obstetrics and Gynecology*.

[32] Laskowska M., Laskowska K., Terbosh M., Oleszczuk J. (2013). A comparison of maternal serum levels of endothelial nitric oxide synthase, asymmetric dimethylarginine, and homocysteine in normal and preeclamptic pregnancies. *Medical Science Monitor : International Medical Journal of Experimental and Clinical Research*.

[33] Kim Y. J., Park H. S., Lee H. Y. (2006). Reduced L-arginine level and decreased placental eNOS activity in preeclampsia. *Placenta*.

[34] Maas R., Boger R. H., Schwedhelm E. (2004). Plasma concentrations of asymmetric dimethylarginine (ADMA) in Colombian women with pre-eclampsia. *Journal of the American Medical Association*.

[35] Noorbakhsh M., Kianpour M., Nematbakhsh M. (2013). Serum levels of asymmetric dimethylarginine, vascular endothelial growth factor, and nitric oxide metabolite levels in preeclampsia patients. *ISRN Obstetrics and Gynecology*.

[36] Perticone F., Sciacqua A., Maio R. (2005). Asymmetric dimethylarginine, L-arginine, and endothelial dysfunction in essential hypertension. *Journal of the American College of Cardiology*.

[37] Grafka A., Łopucki M., Karwasik-Kajszczarek K., Stasiak-Kosarzycka M., Miturski A., Dzida G. (2016). Study of the role of L-arginine in the diagnosis of pregnancy-induced hypertension.

[38] Noris M., Todeschini M., Cassis P. (2004). L-Arginine depletion in preeclampsia orients nitric oxide synthase toward oxidant species. *Hypertension*.

[39] Khan A., Dawoud H., Malinski T. (2018). Nanomedical studies of the restoration of nitric oxide/peroxynitrite balance in dysfunctional endothelium by 1,25-dihydroxy vitamin D_3_ – clinical implications for cardiovascular diseases. *International Journal of Nanomedicine*.

[40] Yzydorczyk C., Armengaud J. B., Peyter A. C. (2017). Endothelial dysfunction in individuals born after fetal growth restriction: cardiovascular and renal consequences and preventive approaches. *Journal of Developmental Origins of Health and Disease*.

[41] Isik D. U., Bas A. Y., Demirel N. (2016). Increased asymmetric dimethylarginine levels in severe transient tachypnea of the newborn. *Journal Of Perinatology*.

[42] Vukosavljevic N., Jaron D., Barbee K. A., Buerk D. G. (2006). Quantifying the l-arginine paradox in vivo. *Microvascular Research*.

[43] Shin S., Mohan S., Fung H. (2011). Intracellular L-arginine concentration does not determine NO production in endothelial cells: implications on the “L-arginine paradox”. *Biochemical and Biophysical Research Communications*.

[44] Hallemeesch M. M., Lamers W. H., Deutz N. E. (2002). Reduced arginine availability and nitric oxide production. *Clinical Nutrition*.

[45] Chamy V. M., Lepe J., Catalán Á., Retamal D., Escobar J. A., MADRID E. M. (2006). Oxidative stress is closely related to clinical severity of pre-eclampsia. *Biological Research*.

[46] Zheng J. J., Wang H. O., Huang M., Zheng F. Y. (2016). Assessment of ADMA, estradiol, and progesterone in severe preeclampsia. *Clinical and Experimental Hypertension*.

[47] Odame E. A. (2015). *Angiogenic factors and oxidative stress biomarkers in gestational hypertension and preeclampsia*.

[48] Rapocka M., Kowalska J., Blumska-Hepner K., Markwitz W., Breborowicz G. H. (2007). The effect of L-arginine on fetal outcome in IUGR fetuses. *Archives of Perinatal Medicine*.

[49] Dera A., Rapocka M., Kowalska J., Markwitz W., Nycz P., Breborowicz G. H. (2007). The effect of L-arginine treatment on the neonatal outcome from pregnancies complicated by intrauterine growth restriction and gestational hypertension. *Archives of Perinatal Medicine*.

[50] Powers R. W., Catov J. M., Bodnar L. M., Gallaher M. J., Lain K. Y., Roberts J. M. (2008). Evidence of endothelial dysfunction in preeclampsia and risk of adverse pregnancy outcome. *Reproductive Sciences*.

[51] Asare L. (2017). Serum homocysteine, vitamin B_12_ and folate in Ghanaian women with hypertensive diorders of pregnancy.

[52] Alexander G. R., Himes J. H., Kaufman R. B., Mor J., Kogan M. (1996). A United States national reference for fetal growth. *Obstetrics and Gynecology*.

