# Supplementary material for: Altered Bioavailability of Nitric Oxide and L-Arginine Is a Key Determinant of Endothelial Dysfunction in Preeclampsia
Source: Biomed Res Int. 2020 Oct 22;2020:3251956. doi: 10.1155/2020/3251956 (PMC7599412; doi:10.1155/2020/3251956)
Supplement: Supplementary 1 — Research questionnaire. [file 3251956.f1.docx]

**RESEARCH QUESTIONNAIRE**

**EVALUATION OF ENDOTHELIAL DYSFUNCTION AND PREGNANCY OUTCOMES IN PREECLAMPSIA**

**QUESTIONNAIRE #: ………………** DATE OF VISIT: …/……/…….

***Kindly fill section A, B and C of this questionnaire***

**SECTION A: SOCIODEMOGRAPHIC CHARACTERISTICS**

1. Please indicate your name …………………………………………………………………….
2. Please indicate your age (years)…………………………………………………………………
3. Please indicate your residential area……………………………………………………………
4. Please indicate your telephone number………………………………………………………….
5. What is your marital status? Married [ ] Single [ ] Divorced [ ] Widowed [ ]
6. Please indicate your level of education? None [ ] Primary [ ] Junior High [ ] Senior High [ ] Tertiary [ ] Other…………………………………………………………… (Please specify)
7. What is your ethnic group? Ewe [ ] Ga [ ] Akan [ ]

Other……………………….……………………………………………… (Please specify)

1. Are you employed? Yes [ ] No [ ]

If yes, name your occupation………………………………………………………………….

**SECTION B: OBSTETRIC AND MEDICAL HISTORY**

1. Have you been pregnant before? Yes [ ] No [ ]

*Skip to question 12 if answer to question 9 is* ***NO***.

1. How many times have you been pregnant? ……………………………………………..
2. Please indicate the following if applicable:
3. Number of live births; ………………………
4. Number of miscarriages: ……………………
5. Number of still births ……………….............
6. Number of abortions ……………………...…
7. Number of preterm deliveries ………………...
8. Number of multiple births ………………….
9. What is your parity? ………………………………………………………………………….
10. What is your gravidity? ………………………………………………………………………
11. Do you have any of the underlisted health conditions?

| 1. Pre-eclampsia | Yes [ ] No [ ] |
| --- | --- |
| 1. Heart disease | Yes [ ] No [ ] |
| 1. Hypertension | Yes [ ] No [ ] |
| 1. Kidney disease | Yes [ ] No [ ] |
| 1. Liver diseases | Yes [ ] No [ ] |
| 1. Diabetes mellitus | Yes [ ] No [ ] |

1. Have you ever been diagnosed of any of the above condition prior to this pregnancy? Yes [ ] No [ ]

If yes, please specify ………………………………..………………………………………

1. Are you currently on any medications or injections? Yes [ ] No [ ]

If yes, list them? ………..………………………………………………..………………….

1. Do you have any other health complication? Yes [ ] No [ ]

If yes, please specify ………………………………..………………………………………

1. Do you have any history of alcoholism? Yes [ ] No [ ]

If yes, how long did you drink? ……………………………………………………………..

1. Have you ever smoked? Yes [ ] No [ ]

If yes, how long did you smoke? ……………………………………………………………

1. Have you ever used any contraceptive? Yes [ ] No [ ]

If yes,

1. Specify the type of contraceptive? ………………………………………………………...
2. How long have you used it? ………………………………………………………….....
3. Have long have you stopped using it before your recent pregnancy? ………………………………………………………………………….

**SECTIO N C: FAMILY HISTORY**

1. Do you have family history of any of the following conditions?

| 1. Pre-eclampsia | Yes [ ] No [ ] |
| --- | --- |
| 1. Heart disease | Yes [ ] No [ ] |
| 1. Hypertension | Yes [ ] No [ ] |
| 1. Kidney disease | Yes [ ] No [ ] |
| 1. Liver diseases | Yes [ ] No [ ] |
| 1. Diabetes mellitus | Yes [ ] No [ ] |
| 1. Multiple pregnancies | Yes [ ] No [ ] |

**SECTION D: This section is to be filled by the researcher.**

1. **OBSTETRIC HISTORY**
2. Expected date of delivery……………………………………………
3. Gestational age at the time of sampling? …………………… . (weeks)
4. Gestational age at sampling …………………………………… (weeks)
5. Weight at first antenatal visit…………………………………… (Kg)
6. Weight at sampling …………….………………………………(Kg)
7. Height ………………………………………...………………. (cm)
8. First blood pressure reading: ………………………………….. (mmHg)
9. Second blood pressure reading: ………………………………. (mmHg)
10. **INTRAPARTUM AND POSTPARTUM CHARACTERISTIC**
11. Estimated fetal weight (EFW) ……………………………………(grams)
12. Gestational age for which EFW was determined…………………. (weeks)
13. Mode of delivery: Vaginal [ ] Spontaneous [ ] Induced [ ]
14. Live birth: Yes [ ] No [ ]
15. Caesarean section: Emergency [ ] Elective [ ]
16. Status of delivery: Term [ ] Preterm [ ]
17. Stillbirth: Fresh [ ] Macerated [ ]
18. Birth weight …………………………………… (grams)
