# Supplementary material for: Altered Bioavailability of Nitric Oxide and L-Arginine Is a Key Determinant of Endothelial Dysfunction in Preeclampsia
Source: Biomed Res Int. 2020 Oct 22;2020:3251956. doi: 10.1155/2020/3251956 (PMC7599412; doi:10.1155/2020/3251956)
Supplement: Supplementary 2 — Smoothed percentiles of estimated birth weight (grams) for gestational age [52]. [file 3251956.f2.docx]

**Smoothed percentiles of estimated birth weight (grams) for gestational age**


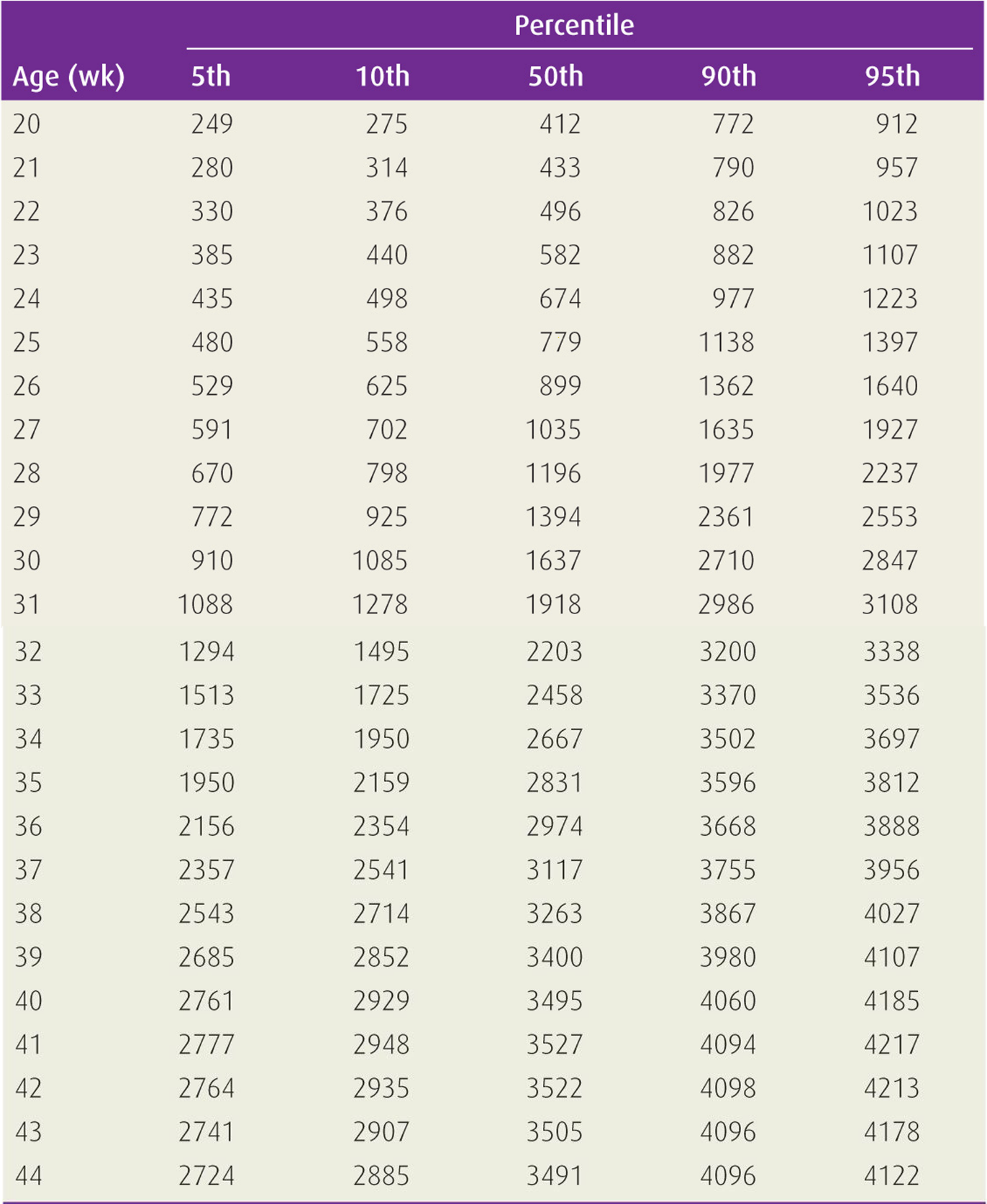


***Source***: Alexander GR, Himes JH, Kaufman RB, Mor J, Kogan M. A United States national reference for fetal growth. Obstetrics and gynecology. 1996;87(2):163-8 ([49](#_ENREF_49)).
